# Cytotoxic drug sensitivity of Epstein-Barr virus transformed lymphoblastoid B-cells

**DOI:** 10.1186/1471-2407-6-265

**Published:** 2006-11-13

**Authors:** Laszlo Markasz, György Stuber, Emilie Flaberg, Åsa Gustafsson Jernberg, Staffan Eksborg, Eva Olah, Henriette Skribek, Laszlo Szekely

**Affiliations:** 1 Department of Microbiology, Tumor and Cell Biology (MTC) and Center for Integrative Recognition in the Immune System (IRIS), Karolinska Institute, Box 280 S-17177 Stockholm, Sweden; 2 Department of Pediatrics University of Debrecen, Medical and Health Science Center, Debrecen, Hungary; 3 Department of Pediatrics, Karolinska Institutet, Karolinska University Hospital, Huddinge, Sweden; 4 Karolinska Pharmacy, and Department of Woman and Child Health, Childhood Cancer Research Unit, Karolinska Institutet, Karolinska University Hospital, Stockholm, Sweden

## Abstract

**Background:**

Epstein-Barr virus (EBV) is the causative agent of immunosuppression associated lymphoproliferations such as post-transplant lymphoproliferative disorder (PTLD), AIDS related immunoblastic lymphomas (ARL) and immunoblastic lymphomas in X-linked lymphoproliferative syndrome (XLP). The reported overall mortality for PTLD often exceeds 50%. Reducing the immunosuppression in recipients of solid organ transplants (SOT) or using highly active antiretroviral therapy in AIDS patients leads to complete remission in 23–50% of the PTLD/ARL cases but will not suffice for recipients of bone marrow grafts. An additional therapeutic alternative is the treatment with anti-CD20 antibodies (Rituximab) or EBV-specific cytotoxic T-cells. Chemotherapy is used for the non-responding cases only as the second or third line of treatment. The most frequently used chemotherapy regimens originate from the non-Hodgkin lymphoma protocols and there are no cytotoxic drugs that have been specifically selected against EBV induced lymphoproliferative disorders.

**Methods:**

As lymphoblastoid cell lines (LCLs) are well established *in vitro *models for PTLD, we have assessed 17 LCLs for cytotoxic drug sensitivity. After three days of incubation, live and dead cells were differentially stained using fluorescent dyes. The precise numbers of live and dead cells were determined using a custom designed automated laser confocal fluorescent microscope.

**Results:**

Independently of their origin, LCLs showed very similar drug sensitivity patterns against 29 frequently used cytostatic drugs. LCLs were highly sensitive for vincristine, methotrexate, epirubicin and paclitaxel.

**Conclusion:**

Our data shows that the inclusion of epirubicin and paclitaxel into chemotherapy protocols against PTLD may be justified.

## Background

Development of malignant B-cell lymphomas after organ transplantation is a significant complication arising as a side effect of the immunosuppression required for successful graft survival. The oncogenic Epstein-Barr virus (EBV) is the etiologic agent in the posttransplant lymphoproliferative disorder (PTLD) and AIDS related immunoblastic lymphomas (ARL) [1]. The reported overall mortality for PTLD often exceeds 50% [2,3]. The prognosis for PTLDs occurring after bone marrow transplantation is even worse [4,5]. Male patients with the rare inherited X-linked lymphoproliferative syndrome, showing specific immune defect against EBV infection, also often succumb to EBV induced malignant lymphomas [6].

EBV is a ubiquitous human herpesvirus that persists for life. Primary EBV infection can lead to mononucleosis (IM) in adolescence and in adults, manifested by a massive expansion of B cells. EBV-encoded transformation-associated proteins drive the proliferation of B lymphoblasts in IM, in PTLDs and in immunodeficiency syndrome-associated immunoblastic lymphomas. The EBV transformed cells express nine latency-associated viral proteins: EBNA1-6, LMP-1, -2A and -2B. This latency program is referred to as the type III latency. The same latency program is present in the *in vitro *proliferating lymphoblastoid cell lines (LCLs), generated by infection of normal human B cells with EBV. The fraction of B cells that is susceptible to *in vitro *transformation can be anything between 10% and 100% [7].

EBV drives the proliferation of human B cells *in vitro *and during primary infection *in vivo*. Strong T cell-mediated immune responses have been documented against EBV encoded latent proteins and a wide range of HLA class I molecules with EBV originated peptide epitopes have been identified [8-10]. EBV associated lymphoproliferative disease can develop only in the absence of a competent cytotoxic T cell immune surveillance. EBV associated lymphoproliferative disease may disappear upon treatment restoring the immune response against EBV-infected B cells.

Historically LCLs were often regarded as non-tumorigenic in immunosupressed mice upon subcutaneous inoculation, especially in comparison with highly tumorigenic Burkitt's lymphomas. However intraperitoneal inoculation regularly leads to development of generalized lymphomas with multiorgan involvement. SCID mice inoculated intraperitoneally with peripheral blood lymphocytes (PBL) from EBV-seropositive donors or with human LCLs, develop EBV-induced human lymphoproliferations within a few weeks. These lymphomas are classified as immunoblastic lymphomas, often with plasmacytoid features [11]. Histologically the PBL derived human-SCID tumors very much resemble the EBV positive large cell lymphomas of immunosuppressed patients [12]. The tumors of the immunocompromised patients or the experimental tumors growing in immunodefective mice as well as the *in vitro *growing LCLs show very similar phenotypes. All three express the same spectrum of cell surface markers, B cell activation antigens and adhesion molecules. All three have normal karyotype and show identical viral gene expression patterns.

The risk of PTLD has been found to depend upon the type of the transplanted organ, the immunosuppressive regimen, the age, the underlying illness and the EBV status of the recipient at the time of transplantation. The estimated incidence of PTLD ranges from 1–4% after renal transplantation to 19% after intestinal transplantation. In bone marrow allograft recipients PTLD is relatively uncommon (1%) [13,14] except for when certain high risk regimes, such as in vitro T-cell depletion (TCD) are used, when the risk may rise to 30%[4,15] PTLD following allogenic stem cell transplantation usually derives from donor lymphocytes. The risk of PTLD is greater if the host is EBV-seronegative at the time of transplantation and/or if there is a mismatch between the donor and recipient HLA types [1].

No controlled studies have been performed in the management of PTLD and most of the recommendations for therapy come from small cohorts at single institutions [1]. The relative importance of T cell impairment, EBV and clonal proliferation has led to the following strategies: reduction of immunosuppression or prophylactic restoration of T-cell immunity [16], antiviral therapy and chemotherapy. Reducing the immunosuppression leads to complete and durable remission of PTLD [17] for 23–50% of patients after organ transplantation (SOD) but will not be efficient in the BMT setting. Reduction of immunosuppression is frequently the first therapeutic step, and patients who have had organ rejection have a much poorer prognosis.

For secondary EBV lymphomas post BMT none of the above regimens will suffice, except when specifically restoring EBV specific immunity [16]. Anti-B-cell monoclonal antibodies are an effective therapy for PTLD. A combination of anti CD21 and anti-CD24 antibodies were used. However these antibodies are not commercially available and interest has therefore recently turned towards Rituximab, a humanized monoclonal antibody against the pan-B cell marker CD20. The response rate of Rituximab treatment is 65% with a relapse rate of 18% [18,19].

In cases that are not responding to the above mentioned therapy, chemotherapy is used as the second or third line of option.

The most frequently used chemotherapy protocols are the anthracycline based CHOP (cyclophosphamide, doxorubicin, vincristine and prednisolone) and VAPEC-B (doxorubicin, etoposide, cyclophosphamide, methotrexate, bleomycin, and vincristine) [20]. In the study of Muti *et al. *17 of 40 PTLD patients were treated with a combination of reduced immunosuppression and chemotherapy (CHOP, VACOP-B – cyclophosphamide, vincristine, bleomycin, prednisolone, with increasing doses of doxorubicin and etoposide- or DHAP -cisplatinum, cytosine-arabinoside, dexamethasone) [21]. Remission rate for anthracycline-based combination therapy is 69%.

PTLD patients are exposed to a high mortality risk due to chemotherapy treatment related toxicity [1].

The side effects of a drug are the limiting factor in determining the dose. Increasing the dose of a drug leads to a more frequent appearance of side effects. Chemotherapeutic agents can also compromise the survival of the graft. Drugs that are effective even at low doses could therefore, not only give fewer side effects, but also provide a better graft survival.

The aim of this study was to find the most effective cytotoxic drugs against EBV-induced lymphoproliferations. We carried out over 6500 cell survival and proliferation assays, on single cell level, in order to establish the pattern of *in vitro *efficacy of the most commonly used cytotoxic drugs against EBV-transformed lymphoblastoid cells.

## Methods

### Cell lines and culture conditions

17 lymphoproliferative cell lines (LCLs) were used in the present study. 980215, 031016, 040113, 051018, JAK, LP, MIN, AF, GK, FUR, HA, VMB were established by *in vitro *infection of normal B lymphocytes, from different healthy donors, with the B95.8 strain of EBV. These cell lines were expanded in IMDM (Sigma) medium supplemented with 10% fetal calf serum (FCS). LSspontan is a spontaneous LCL established from a healthy donor. IARC171 was established using B95.8 virus infection of normal lymphocytes from a patient with Burkitt's lymphoma. TR was established from B cells of an XLP patient, with deletion in the SAP gene. The cell line IB4 contains an integrated EBV genome.

During the time of experiment the LCLs were cultured in IMDM (Sigma) supplemented with 20% FCS (Sigma), 100 mmol L-glutamine (Sigma) and 80 μg/ml gentamicin (Sigma). Cell suspensions were grown in a humidified incubator at 37°C in an atmosphere containing 5% CO_2_. Cell counts were adjusted to an optimal concentration of 1 × 10^6 ^cells/ml and the cells were fed twice a week. The absence of mycoplasma contamination was assured by regular monitoring with Hoechst 33258 staining.

### Drugs

For the *in vitro *drug sensitivity test 29 drugs were used (summarized in Table 1). All the drugs were dissolved in 50% dimethyl sulfoxide (DMSO) and printed on 384 well plates, using a high density metal pin array (with 50 nl replica volumes) in Biomek 2000 fluid dispenser robot (Beckman). The same robot was used to generate the drug masterplates containing the triplicates of four different drug dilutions using a single tip automatic dispenser head.

The highest drug concentration was selected as the physicochemical maximally achievable drug concentration at 600 times dilution (50 nl drug in 30 μl suspension assay volume)

The Ratio of Maximum Achieved Plasma Concentration (RMAPC) was determined for each drug concentration in order to compare the *in vitro *tested concentrations to the *in vivo *maximally achieved plasma concentration (Cmax) levels.

RMAPC was calculated according to the following equation:

RMAPC = *in vitro *tested concentration/Cmax

### In vitro drug sensitivity assay

In vitro drug sensitivity of LCLs was assessed using a 3-day cell culture on microtiter plates. 28 drugs were tested, each at 4 different concentrations in triplicates on 384-well plates (Greiner). Each well was loaded with 30 μl cell suspension containing 3000 cells. After three days of incubation the live and dead cells were differentially stained using fluorescent VitalDye (Biomarker, Hungary). The exact number of live and dead cells was determined using a custom designed automated laser confocal fluorescent microscope (a modified Perkin-Elmer UltraView LCI system) at the Karolinska Institute visualization core facility (KIVIF). The images were captured using the computer program QuantCapture 4.0 and the live and dead cells were identified and individually counted using the program QuantCount 3.0. Both programs were developed at the KIVIF using OpenLab Automator programming environment (Improvision). 15 control wells, that were used to determine the control cell survival (CCS), contained cells with only culture medium and 50 nl DMSO without drugs, 5 wells contained cells with culture medium alone. Comparing the two types of control wells no toxic effect of DMSO could be seen. Mean cell survival (MCS) was determined from the average of cell survival of all 17 LCLs.

## Results

### Comparison of the drug sensitivity pattern of the different LCLs

The 17 different LCLs that were tested in the present study represent a large variety of cells with different origins and *in vitro *history. The investigation included cell lines with several years of continuous *in vitro *culturing along with freshly established transformed B-cell cultures of only 3 weeks of age. Many of the lines were transformed by the B95-8 strain of EBV and some of them were spontaneous outgrowths driven by the donors own virus. Among the lines, we had LCLs from healthy donors, from XLP patients or lines established from the normal B-cells of Burkitt's lymphoma patients. We tested lines with both episomal and integrated EBV genome.

**Figure 1:**
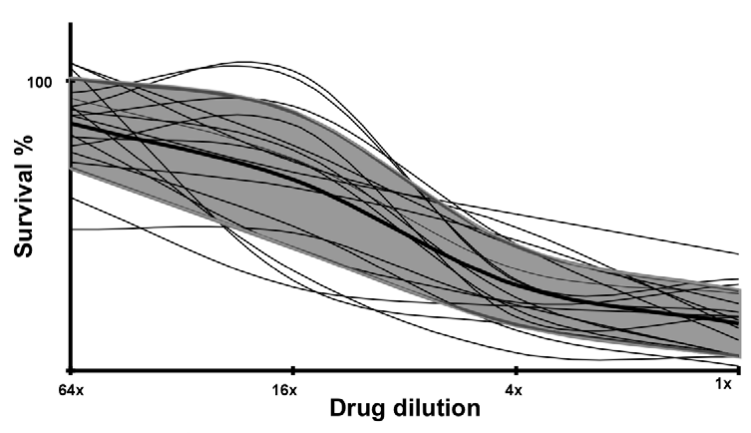
**Drug sensitivity pattern of 17 lymphoblastoid cell lines for epirubicin**. The mean value of survival of LCLs is represented by the black curve, ± SD value is marked with grey. 1× : highest concentration, 16×: 16 times dilution of the highest concentration etc.

**Figure 2:**
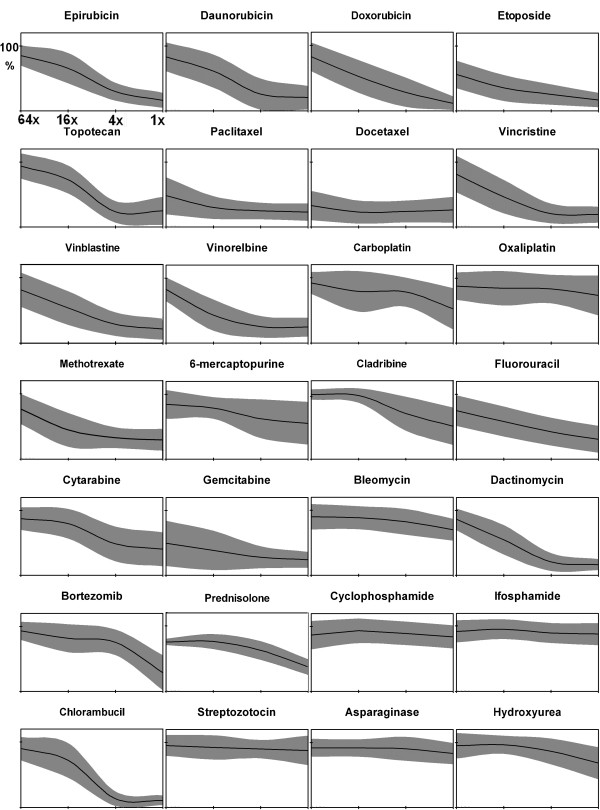
**Mean values and standard deviations of drug sensitivity of 17 lymphoblastoid cell lines for 28 different cytostatic drugs**. y axis: fraction of surviving cells 0–100% x axis: drug dilutions (1× represents the highest concentration).

Remarkably, the LCLs of different origins showed a very similar sensitivity pattern against the different drugs. This phenomenon is illustrated in Figure 1, which summarizes the survival curves of all 17 LCLs against the four different concentrations of epirubicin. Each individual thin curve represents the average of three independent measurements. The thick line is the mean of all curves, the Mean Cell Survival (MCS). The grey shaded area marks the ± standard deviation (SD). Figure 2 summarizes the Mean Cell Survival curve along with the standard deviations for 28 drugs again emphasizing the very similar response of the different cell lines. The values for MG132 are represented only in Table 2.

### Identification of highly effective and non-effective drugs

**Figure 3:**
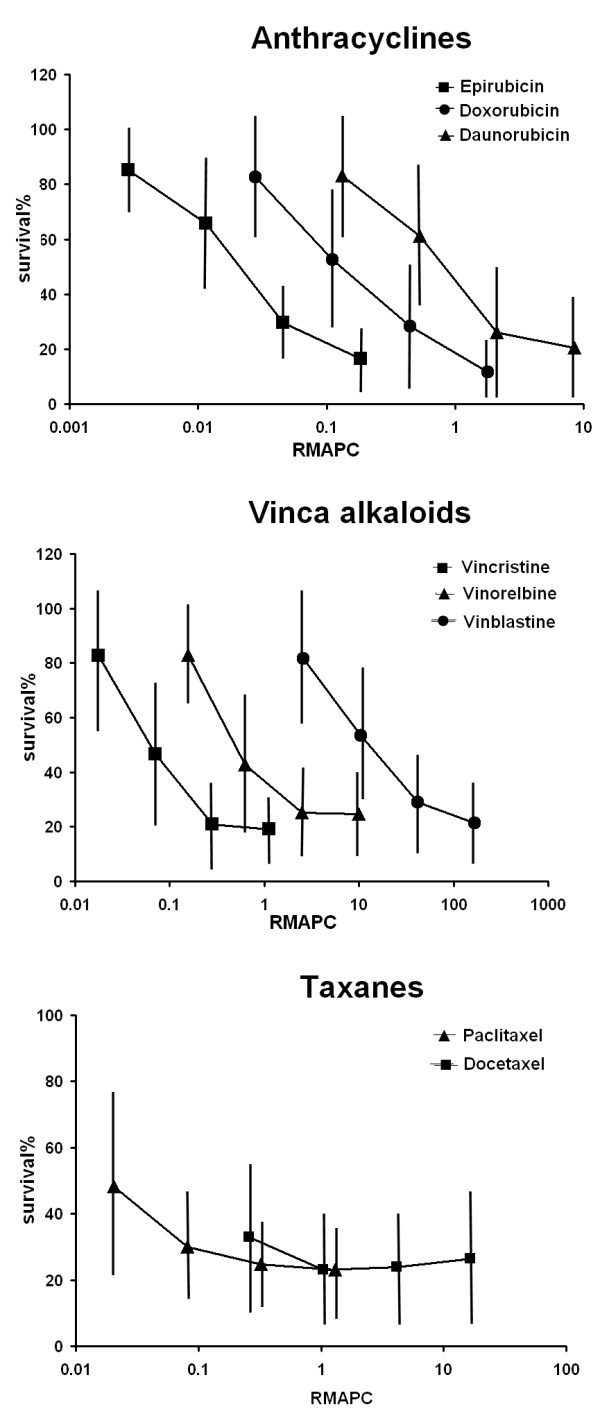
Drug sensitivity of LCLs for anthracyclines, vinca alkaloids and taxans.

**Figure 4:**
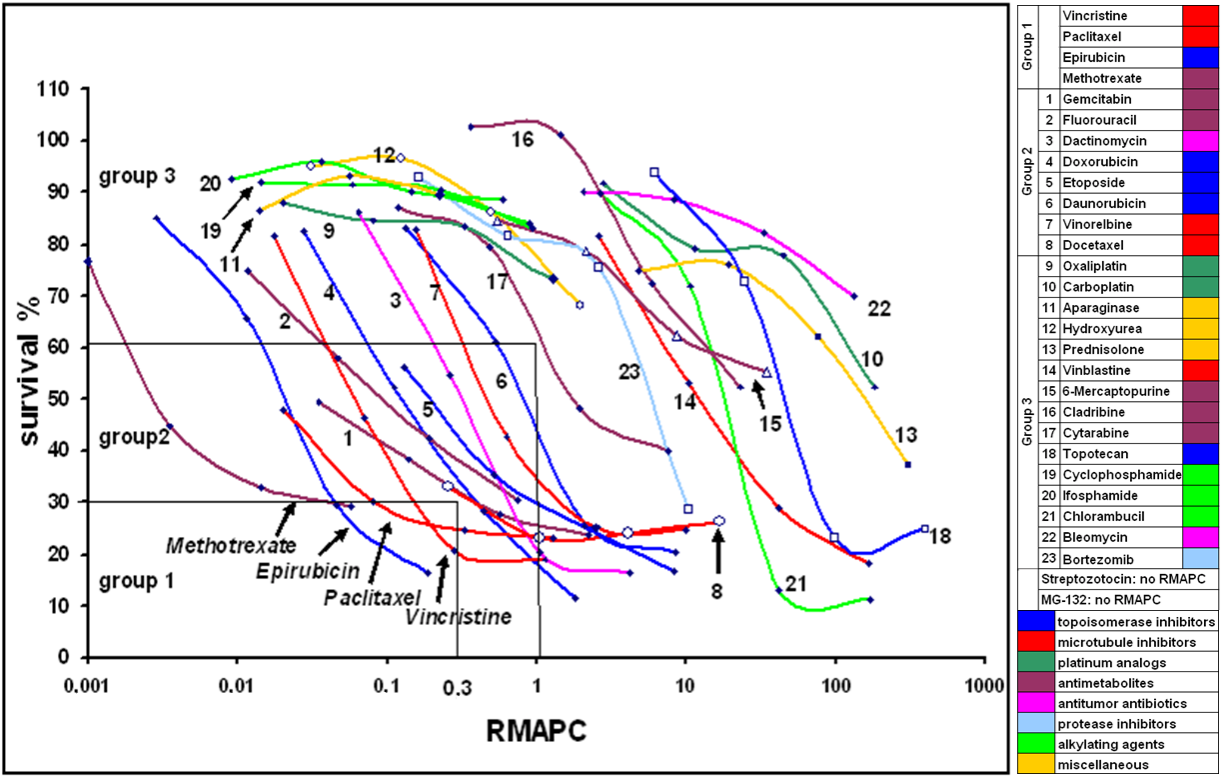
**Drug sensitivity mean values of 17 lymphoblastoid cell lines plotted against the Ratio of Maximum Achieved Plasma Concentration (RMAPC) values**. The different drug families are labelled with different colours. The highly effective (Group 1) and partially effective (Group 2) drugs are identified.

The starting concentrations of the dilution series (1×, 4×, 16×, 64×) for the individual drugs were initially determined based on the solubility of the different agents. To make the cell survival data comparable with each other, we first compiled the known Maximum Achieved Plasma Concentration data (Cmax) for all 28 drugs stated from the literature (Table 1-column 5). Dividing the actual drug concentrations in the dilution series with the Cmax of the particular drug yielded the Ratio of Maximum Achieved Plasma Concentration (RMAPC). The calculated values of RMAPC for all the drugs are summarized in Table 2 along with MCS and ± SD values. Plotting the cell survival against the RMAPC values allowed a direct comparison of the effectiveness of different drugs. Figure 3 shows the comparison of the effectiveness of the members of three different drug families: the anthracyclines, vinca alcaloids and taxans. Figure 4 summarizes the mean cell survival curves for all the drugs along a common RMAPC axis. Based on their effectiveness on LCLs, we have divided the drugs into three different groups:

Group 1: The drugs were considered to be highly effective if the mean cell survival (MCS) was below 30% at RMAPC 0.3 or below that.

Group 2: The drug was partially effective if MCS was under 60% and RMAPC was between 0.3 and 1.

Group 3: The drug was ineffective, e.g. the LCLs were resistant to the drug, if MCS was above 60% or RMAPC was above 1.

We found that the four most effective drugs against LCLs were: **vincristine, paclitaxel, methotrexate and epirubicin **(Group 1).

Gemcitabin, cytosine-arabinoside, doxorubicin, fluorouracil, dactinomycin, docetaxel, daunorubicin, etoposide, vinorelbine was rated as partially effective (Group 2.)

Most LCLs were resistant to cyclophosphamide, asparaginase, topotecan, oxaliplatin, bleomycin, 6-mercaptopurin, hydroxyurea, cladribine, chlorambucil, carboplatin, bortezomib, cytarabine, prednisolone and vinblastine (Group 3). Almost no drug response could be seen with oxaliplatin, bleomycin, cyclophosphamide, asparaginase, hydroxyurea and ifosphamide whereas vinblastine, chlorambucil, prednisolone and topotecan were effective, but only at very high concentrations, well above the maximum achievable plasma concentrations.

Although the Group 3 drugs were not effective against LCLs, these drugs show concentration dependent growth inhibitory effects on other cell lines or primary tumors (data not shown).

No RMAPC could be determined for streptozocin because no pharmacokinetical trials with established Cmax plasma levels were found. RMAPC was also not available for MG132 because it has not been used clinically. Streptozocin had no effect on survival at the concentrations used in the study. Although at the highest concentrations the proteasome inhibitor MG132 effectively decreased the survival of the cells, its clinically licensed functional homologue, bortezomib, was not effective.

An alternative way to calculate relationship between the *in vitro *drug concentrations and the *in vivo *used values is to use the area under curve (AUC) values of the individual drugs. To compare AUC values with the RMAPC data the following formula was used:

*in vitro *used concentration (μg/ml) × 72 h/AUC (μgxh/ml) *in vivo*.

*In vivo *AUC levels from the literature are summarized in Table 1. Using the formula for representing the mean cell survival curves for all the drugs along a common "ratio of AUC" axis, carboplatin, methotrexate and dactinomycin showed higher efficacy in comparison to RMAPC figures whereas the sensitivity order for the other drugs or for the group classifications did not change.

## Discussion

The presented data suggest that many EBV transformed B-cell lines share a common cytotoxic drug sensitivity profile independent of their origin. This profile does not change even after many years of *in vitro *culturing. EBV appears to be the necessary and sufficient etiological agent behind the malignant immunoblastic B-cell lymphoproliferations in immunosuppressed patients. In all these cases the EBV encoded, latency associated viral proteins drive the cell proliferation without any obvious need for additional genetic changes. The phenotype of these tumors closely resembles the *in vitro *growing LCLs and the experimentally induced tumors that appeared upon intraperitoneal implantation of LCLs into SCID mice [11]. Considering the phenotypic and karyotypic stability of EBV transformed B-cells, our data raises the hope that PTLDs, AIDS associated CNS lymphomas and XLP associated lymphoproliferations may show similar patterns of drug sensitivity as the ones that we have established in the present study on a cohort of diverse LCLs.

Sugimoto et al. suggested that EBV mediated transformation is a two step process where, after prolonged passage, the cells may become aneuploid, accumulate p53 mutations, down regulate the p16/retinoblastoma protein pathway and become tumorigenic upon subcutaneous inoculation into nude mice [7]. Some of the cell lines that we have examined were kept in continuous culture over five years (IARC171, LSspontan, 980215). These cells are still euploid and show low soft agar clonability. The oldest line IARC171, has wild type p53, is non-tumorigenic upon subcutan inoculation but produces generalized immunoblastic lymphomas in SCID mice after intraperitoneal inoculation. The findings that PTLD can arise very rapidly in post-transplant patient together with the data that freshly EBV infected B-cells can grow into generalized immunoblastic lymphomas within a few weeks, implies that the development of PTLD does not require aneuploidy. Sawada *et al. *reported that LCLs with negative or low telomerase activity and normal karyotypes are more sensitive against certain drugs, than LCLs with a high telomerase activity and abnormal karyotypes [22]. This data together with our present findings may suggest that rapidly emerging PTLDs may show different drug sensitivity pattern from EBV positive aneuploid diffuse large cell B-cell lymphomas that arise for example in AIDS patients in sub-Saharan Africa.

Our data shows that euploid LCLs are particularly sensitive for anti-microtubule drugs and anthracyclines. Although all LCLs showed a dose dependent increase of cytotoxicity when treated with different members of these two drug families, only vincristine and paclitaxel as well as epirubicin and doxorubicin were considered to be effective when the used *in vitro *drug concentrations were compared to the maximum achievable plasma concentrations.

Alkylating agents, such as cyclophosphamide and ifosphamide were not effective on LCLs. This might be explained by the fact that both of these compounds are pro-drugs that have to be converted into active metabolites by the liver *in vivo*. Prednisolone was active but only in very high concentrations.

## Conclusion

Vincristine and methotrexate are included in the frequently used CHOP and VAPEC-regimes, but no data could be found for the clinical use of epirubicin and paclitaxel for the treatment of PTLD or other EBV induced lymphoproliferations. Our results suggest that the inclusion of epirubicin and paclitaxel into chemotherapy protocols against PTLD may be justified.

## Competing interests

The author(s) declare that they have no competing interests.

## Authors' contributions

The project was conceived and designed by LS. The experiments were mainly carried out and/or coordinated by LM. GS took part in cell culturing, and preparation of the microtiter plates for in vitro drug sensitivity assays. EF was responsible for measuring the plates using the automated laser confocal fluorescent microscope. LS and EF wrote the computer programs QuantCapture 4.0 and QuantCount 3.0. LM and HS analysed and interpreted the data. SE made comparable the in vitro results with the in vivo data. AGJ, EO, HS together with the other authors have been involved in the planning of the experimental details, and the drafting and critical reading of the manuscript. All authors read and approved the final manuscript.

## Pre-publication history

The pre-publication history for this paper can be accessed here:



## Figures and Tables

**Table 1:** Used drugs, Cmax, AUC levels

**Drug groups**	**Drug name**	**Brand name, company**	***In vitro *used concentrations (μg/ml)**	**Cmax Value (μg/ml)**	***In vivo *dose (Cmax)**	**AUC (μg × h/ml)**	***In vivo *dose (AUC)**	**Reference**
anthracyclines	epirubicin	Epirubicin Meda, Meda	0.026–1.66	9	120 mg/m^2^	2.412	90 mg/m^2^	(Fogli et al. 2002) [23]
	daunorubicin	Cerubidin^®^, Aventis Pharma	0.065–4.166	0.49	0.95 mg/kg in 45 min	1.2786	1.5 mg/kg	(Andersson et al. 1979) [6]
	doxorubicin	Doxorubicin Teva, Teva	0.026–1.66	0.93	50 mg/m^2^	0.82464	50 mg/m^2^	(Toffoli et al. 2004) [24]
epipodophyllotoxin	etoposide	Sigma	0.325–20.833	2.5	53 mg/m^2^/day	5.06	100 mg/m^2^/day	(Minami et al. 1995) [25] (Gruber et al.1995) [26]
camptothecins	topotecan	Hycamtin^®^, GlaxoSmithKline	0.052–3.33	0.0084	2.3 mg/m^2^/day	0.0196	1.2 mg/m^2^/day	(Gerrits et al. 1999) [27]
taxanes	paclitaxel	Taxol, Orifarm	0.078–5	3.38	175 mg/m^2 ^by 3 h	13.49	175 mg/m^2^	(Fogli et al. 2002) [23]
	docetaxel	Taxotere^®^, Aventis Pharma	0.52–33.33	2	60 mg/m^2^	3.326	85 mg/m^2^	(Rischin et al. 2002) [28]
vinca alkaloids	vincristine	Vincristine Mayne, Mayne Pharma	0.0065–0.416	0.37	1.4 mg/m^2^	0.182	1.32 mg/m^2^	(Terashi et al. 2000) [29] (Desai et al. 1982) [30]
	vinblastine	Velbe^®^, STADApharm	0.013–0.833	0.005	1.5 mg/m^2 ^by 24 h	0.218	1.7 mg/m^2^	(Stewart et al. 1983) [31] (Bates et al.) [32]
	vinorelbine	Navelbine^®^, Pierre Fabre	0.13–8.33	0.83	200 mg/week	0.899	80 mg/m^2^/week	(Zhou et al. 1991) [33] (Freyer et al. 2003) [34]
platinum analogs	carboplatin	Carboplatin Mayne, Mayne Pharma	0.13–8.33	0.046	360 mg/m^2^	348000	360 mg/m^2^	(Ghazal-Aswad et al. 1996) [35]
	oxaliplatin	Eloxatin, Sanofi-Synthelabo	0.065–4.166	3.2	130 mg/m^2^	71.5	130 mg/m^2^	(Gamelin et al. 1997) [36] (Graham et al. 2000) [37]
antifolate	methotrexate	Methotrexate Pharmacia, Pfizer	0.325–20.833	363	8–12 g/m^2^	13200000	12 g/m^2^	(Bacci et al. 2006) [38] (Crews et al. 2004) [39]
purin antagonists	6-mercaptopurine	Sigma	1.085–69.44	2	2.5 mg/kg oral dose	0.2587	85 mg/m^2^	(Chan et al. 1989) [26]
	cladribine	Leustatin, Janssen-Cilag	0.013–0.833	0.0356	5 mg/m^2^	0.1541	5 mg/m^2^	(Albertioni et al. 1998) [40]
pyrimidine antagonists	fluorouracil	Fluorouracil Mayne, Mayne Pharma	0.65–41.66	55.4	400 mg/m^2^	11.59	400 mg/m^2^	(Casale et al. 2004) [41]
	cytarabine	Cytarabine Pfizer, Pfizer	1.302–83.33	10.8	3 g/m^2^	523.4	1 g/m^2^	(DeAngelis et al. 1992) [42] (Gruber et al. 1995) [26]
	gemcitabine	Gemzar, Orifarm	0.781–50	22.3	1000 mg/m^2^	9.3	1000 mg/m^2^	(Fogli et al. 2002) [23]
antitumor antibiotics	bleomycin	Bleomycin Baxter, Baxter	0.39–25*	0.19*	30 IU/day	0.089	8 IU/kg/day	(Broughton et al. 1977) [43] (Peng et al.1980) [44]
	dactinomycin	Cosmegen^®^, MSD	0.006–0.416	0.1	1.5 mg/m^2^	300	1.5 mg/m^2^	(Veal et al. 2005) [45]
protease inhibitors	bortezomib	Velcade^®^, Janssen-Cilag	0.013–0.833	0.08	1.45 mg/m^2^	0.0438	1.45 mg/m^2^	(Papandreou et al. 2004) [46]
	MG132	Sigma	0.13–8.33	**#**				
alkylating agents	cyclophosphamide	Sendoxan, Baxter	0.52–33.33	37.2	50 mg/kg	367	50 mg/kg	(Xie et al. 2006) [47]
	ifosphamide	Holoxan^®^, Baxter	0.52–33.33	56.63	16 g/m^2 ^in 96 h	1827.7	3 g/m^2^/day	(Wright et al. 1995) [48] (Boddy et al. 1996) [45]
	chlorambucil	Sigma	1.302–83.33	0.49	0.2 mg/m^2^	0.883	0.2 mg/m^2^	(GlaxoSmithKline Research Triangle Park) [49]
	streptozotocin	Sigma	0.65–41.66	**¤**				
miscellaneous	asparaginase	Asparaginase Medac, Medac	0.065–4.166*	4.48*	10000 IU/m^2^	0.943	30000 IU/m^2^	(Ho et al. 1981) [50] (Ylikangas et al. 2002) [51]
	hydroxyurea	Sigma	0.65–41.66	21	18 mg/kg	82.49	15 mg/kg	(Gwilt et al. 2003) [52] (Yan et al. 2005) [46]
	prednisolone	Di-Adreson F Aquosum, Organon	1.56–100	0.325	1 mg/kg/day	1.33	1 mg/kg/day	(Rostin et al. 1990) [53]

**Cmax**: maximum plasma level after administration of the clinical dose of the drug

*In case of bleomycin and asparaginase concentrations are in IU/ml

**AUC**: area under curve

#: not used in the clinical practice

¤: no data is available

**Table 2:** The average Mean Cell Survival (MCS) of the 17 LCLs at different drug concentrations, expressed as the Ratio of Maximum Achieved Plasma Concentration (RMAPC)

		64× dilution	16× dilution	4× dilution	1× no dilution		64× dilution	16× dilution	4× dilution	1× no dilution
**Group 1**	**Vincristine**					**Epirubicin**				
	**RMAPC**	0.018	0.071	0.282	1.128	**RMAPC**	0.003	0.012	0.046	0.184
	**MCS**	81.498	46.541	20.776	18.995	**MCS**	85.206	65.736	29.435	16.306
	**SD**	27.995	25.737	15.258	12.209	**SD**	15.548	23.394	13.593	11.314
	
	**Paclitaxel**					**Methotrexate**				
	**RMAPC**	0.020	0.081	0.326	1.303	**RMAPC**	0.001	0.004	0.014	0.057
	**MCS**	48.119	29.999	24.653	23.148	**MCS**	76.618	44.824	32.924	29.314
	**SD**	28.435	17.418	12.869	13.364	**SD**	22.796	25.843	15.590	16.455

**Group 2**	**Gemcitabin**					**Dactinomycin**				
	**RMAPC**	0.035	0.140	0.561	2.242	**RMAPC**	0.066	0.262	1.050	4.200
	**MCS**	49.354	38.411	27.808	23.784	**MCS**	86.206	54.700	20.520	16.465
	**SD**	34.718	30.034	16.945	12.153	**SD**	16.701	22.386	13.642	8.370
	
	**5-Flurouracil**					**Vinorelbine**				
	**RMAPC**	0.012	0.047	0.188	0.752	**RMAPC**	0.156	0.625	2.501	10.005
	**MCS**	74.861	58.001	42.337	30.505	**MCS**	82.833	42.823	25.176	24.683
	**SD**	23.686	22.987	21.370	20.645	**SD**	18.131	23.930	16.506	14.634
	
	**Doxorubicin**					**Daunorubicin**				
	**RMAPC**	0.028	0.112	0.447	1.789	**RMAPC**	0.133	0.531	2.126	8.502
	**MCS**	82.503	52.477	28.292	11.499	**MCS**	83.171	61.159	25.940	20.439
	**SD**	21.753	24.867	22.314	10.301	**SD**	21.886	25.402	23.933	17.961
	
	**Etoposide**					**Docetaxel**				
	**RMAPC**	0.130	0.521	2.083	8.333	**RMAPC**	0.260	1.042	4.166	16.665
	**MCS**	56.281	35.375	25.315	16.778	**MCS**	32.990	23.036	23.866	26.331
	**SD**	21.045	19.983	15.497	11.166	**SD**	22.690	17.587	16.810	19.810

**Group 3**	**Cytarabine**					**6-Mercaptopurine**				
	**RMAPC**	0.121	0.482	1.929	7.716	**RMAPC**	0.543	2.170	8.680	34.720
	**MCS**	87.321	79.450	48.255	40.124	**MCS**	84.450	78.834	62.325	55.419
	**SD**	17.396	22.850	26.423	26.315	**SD**	22.147	16.514	29.820	32.858
	
	**Oxaliplatin**					**Cladribine**				
	**RMAPC**	0.020	0.081	0.325	1.301	**RMAPC**	0.366	1.462	5.850	23.399
	**MCS**	87.951	84.477	83.331	73.511	**MCS**	102.832	101.241	72.497	52.237
	**SD**	21.010	26.545	21.839	30.744	**SD**	8.130	11.160	26.804	29.545
	
	**Asparaginase**					**Chlorambucil**				
	**RMAPC**	0.015	0.058	0.232	0.930	**RMAPC**	2.646	10.586	42.342	169.370
	**MCS**	92.021	91.570	90.371	82.978	**MCS**	89.903	71.879	13.225	11.099
	**SD**	13.718	13.222	18.153	15.919	**SD**	17.578	26.506	10.994	7.527
	
	**Cyclophosphamide**					**Topotecan**				
	**RMAPC**	0.014	0.056	0.224	0.896	**RMAPC**	6.194	24.777	99.107	396.429
	**MCS**	86.670	93.412	89.498	84.041	**MCS**	93.897	72.802	23.250	24.784
	**SD**	21.910	18.833	17.832	17.920	**SD**	19.406	21.275	16.659	21.380
	
	**Ifosphamide**					**Carboplatin**				
	**RMAPC**	0.009	0.037	0.147	0.588	**RMAPC**	2.814	11.257	45.027	180.108
	**MCS**	92.729	95.906	90.101	88.518	**MCS**	91.805	79.175	77.962	52.322
	**SD**	16.694	14.965	15.299	16.677	**SD**	16.670	30.927	22.065	31.295
	
	**Hydroxyurea**					**Bleomycin**				
	**RMAPC**	0.031	0.124	0.496	1.984	**RMAPC**	2.078	8.311	33.245	132.979
	**MCS**	95.118	96.489	86.340	68.134	**MCS**	90.007	88.553	82.168	69.803
	**SD**	19.595	13.933	19.759	24.782	**SD**	19.294	17.678	19.720	16.686
	
	**Vinblastine**					**Streptozotocin**				
	**RMAPC**	2.603	10.413	41.650	166.600	**RMAPC**	no Cmax data was found			
	**MCS**	81.507	53.331	28.885	21.323	**MCS**	95.430	92.335	89.919	87.805
	**SD**	25.921	25.678	17.941	15.889	**SD**	15.828	18.710	14.094	22.637
	
	**Bortezomib**					**MG-132**				
	**RMAPC**	0.162	0.648	2.590	10.361	**RMAPC**	not used in clinical practice			
	**MCS**	93.119	81.671	75.498	28.700	**MCS**	87.345	96.374	88.097	27.045
	**SD**	13.779	21.572	19.617	26.948	**SD**	13.980	17.493	16.974	24.310
	
	**Prednisolone**									
	**RMAPC**	4.8	19.23077	76.92308	307.6923					
	**MCS**	75.55877	76.25595	62.46537	37.23379					
	**SD**	4.647172	11.09091	13.97021	11.65414					

Group 1. Highly effective drugs: RMAPC < 0.3; MCS < 30%

Group 2. Partially effective drugs: RMAPC between 0.3 and 1, MCS < 60%

Group 3. Ineffective drugs: RMAPC > 1 or MCS > 60%

SD: standard deviation in mean cell survival
